# Oxidative Stress Induced Age Dependent Meibomian Gland Dysfunction in Cu, Zn-Superoxide Dismutase-1 (*Sod1*) Knockout Mice

**DOI:** 10.1371/journal.pone.0099328

**Published:** 2014-07-18

**Authors:** Osama M. A. Ibrahim, Murat Dogru, Yukihiro Matsumoto, Ayako Igarashi, Takashi Kojima, Tais Hitomi Wakamatsu, Takaaki Inaba, Takahiko Shimizu, Jun Shimazaki, Kazuo Tsubota

**Affiliations:** 1 Keio University School of Medicine, Department of Ophthalmology, Tokyo, Japan; 2 Tokyo Dental College, Department of Ophthalmology, Chiba, Japan; 3 Department of Advanced Aging Medicine, Chiba University Graduate School of Medicine; Chiba, Japan; University of Florida, United States of America

## Abstract

**Purpose:**

The purpose of our study was to investigate alterations in the meibomian gland (MG) in Cu, Zn-Superoxide Dismutase-1 knockout (*Sod1* ^−/−^) mouse.

**Methods:**

Tear function tests [Break up time (BUT) and cotton thread] and ocular vital staining test were performed on *Sod1* ^−/−^ male mice (n = 24) aged 10 and 50 weeks, and age and sex matched wild–type (+/+) mice (n = 25). Tear and serum samples were collected at sacrifice for inflammatory cytokine assays. MG specimens underwent Hematoxylin and Eosin staining, Mallory staining for fibrosis, Oil Red O lipid staining, TUNEL staining, immunohistochemistry stainings for 4HNE, 8-OHdG and CD45. Transmission electron microscopic examination (TEM) was also performed.

**Results:**

Corneal vital staining scores in the *Sod1* ^−/−^ mice were significantly higher compared with the wild type mice throughout the follow-up. Tear and serum IL-6 and TNF-α levels also showed significant elevations in the 10 to 50 week *Sod1* ^−/−^ mice. Oil Red O staining showed an accumulation of large lipid droplets in the *Sod1* ^−/−^ mice at 50 weeks. Immunohistochemistry revealed both increased TUNEL and oxidative stress marker stainings of the MG acinar epithelium in the *Sod1* ^−/−^ mice compared to the wild type mice. Immunohistochemistry staining for CD45 showed increasing inflammatory cell infiltrates from 10 to 50 weeks in the *Sod1* ^−/−^ mice compared to the wild type mice. TEM revealed prominent mitochondrial changes in 50 week *Sod1* ^−/−^ mice.

**Conclusions:**

Our results suggest that reactive oxygen species might play a vital role in the pathogensis of meibomian gland dysfunction. The *Sod1* ^−/−^ mouse appears to be a promising model for the study of reactive oxygen species associated MG alterations.

## Introduction

Meibomian glands (MG) are large sebaceous glands located in the tarsal plates of the eyelid that secrete a mixture of lipids and proteins called meibum onto the ocular surface, forming a superficial lipid layer that prevents the evaporation of tears [1].

Abnormalities in the MGs cause instability of the tear film, resulting in either chronic irritation of, or damage to, the ocular surface epithelium. Hyposecretion of meibomian lipids (meibum) caused by obstruction of the gland orifices is the most common abnormality and is usually referred to as obstructive meibomian gland dysfunction (MGD). MGD is an age related disease that leads to decreased expression of glandular lipids and an increase in tear instability, leading to dry eye disease and ocular surface abnormalities [1], [2], [3], [4], [5], [6].

It has been reported that the incidence of dry eye is extensive in the Japanese population aged over 60, with MGD being the predominant associated disease process, as evidenced by trans-illumination of the eyelids [7]. The overall prevalence of MGD is 38.9% in the US, but it increases markedly with age, from 0% for subjects under the age of 10 to 67.2% for those over the age of 60 [8].

Aging is associated with an accumulation of reactive oxygen species (ROS) that damage all components of cells, including proteins, lipids, and DNA, leading to age-related chronic diseases [9], [10]. This is believed to be a result of an imbalance between the ROS and antioxidant defense enzymes, including the superoxide dismutase (SOD) family, which includes SOD1, SOD2 and SOD3 enzymes.

Although MGD is the most commonly associated dry eye disease in the elderly that effects the health and well-being of millions of people around the world, its incidence is conceivably underestimated [1]. Due to ethical issues, the mechanism of MGD development, as well as the implications on the ocular surface, the histopathology of the diseased MG have not been studied extensively in human subjects. This has led to studies using appropriate animal models.

Our previous studies showed that mice deficient in Cu, Zn superoxide dismutase (SOD1) to be a suitable animal model for the study of human age related diseases, as the mice display all the features of age related macular degeneration and lacrimal gland dysfunction observed in humans [11], [12]. A recent report showed that mouse MGs undergo age-related changes similar to those identified in humans [13].

In this study, we investigated the histopathology and mechanism of MG alterations in *Sod1* ^−/−^ mice in order to investigate the relationship of the alterations to age dependent dry eye disease.

## Materials and Methods

### Ethics statement

All studies were performed in accordance with the Association for Research in Vision and Ophthalmology (ARVO) Statement for the Use of Animals in Ophthalmic and Vision Research. The study was approved by Tokyo Dental College Ichikawa Hospital Ethics Committee for Animal Research.

### Animals

Twenty four *Sod1* ^−/−^ male mice with C57BL/6 background (14 [10 ws], 10 [50 ws]) and twenty five C57BL/6 strain wild–type (WT) male mice (11 [10 ws], 14 [50 ws]) were examined at 10 and 50 weeks. The *Sod1* ^−/−^ mice were supplied by the Tokyo Metropolitan Institute of Gerontology, while the wild-type C57BL/6 mice were purchased from Japan Clea (Osaka, Japan). *Sod1* ^−/−^ mice were backcrossed to *Sod2^flox/flox14^* for two generations to obtain the *Sod1* ^−/−^ and *Sod2^flox/flox^*. *Sod2 flox flox* backcross do not have the cre gene sequence and the resultant mice lack the SOD2 gene in several tissues including bone, skin, liver, ovaries and erythrocytes as already published by Shimizu et al. [14], [15] It should be noted that there is a possibility that the phenotypes may have arisen spontaneously in the separately breeding colony of *Sod1* ^−/−^ mice. The chances of this occurrence may increase as the number of generations of breeding increases.

### Aqueous tear production measurements

Phenol red–impregnated cotton threads (Zone-Quick, Showa Yakuhin Kako Co., Ltd., Tokyo, Japan) were used to measure aqueous tear production without anesthesia [12]. Briefly, cotton threads were immersed into the tear meniscus in the lateral canthus of the mice eyes for 60 seconds and the length of wetting of the thread was measured in millimeters. Tear production was weight adjusted by dividing the total amount of aqueous tear secretion produced in 60 seconds by weight. Tear production was assessed in the right and left eyes of each mouse on two occasions; first at the age of 10 weeks and the second time at 50 weeks in both *Sod1^−/−^* and WT mice.

### Ocular surface tear film stability and corneal epithelial cell damage evaluation

Tear film stability was measured using the tear film break up time (BUT) test. 2 µl of 0.5% sodium fluorescein was instilled into the conjunctival sac. After blinking, BUT was recorded in seconds three times then averaged to obtain the mean BUT value per eye. Corneal epithelial damage was assessed 2 minutes after the fluorescein dye instillation. Tear film stability and fluorescein staining tests were conducted using a hand-held cobalt blue light slit-lamp biomicroscope (Kowa, Tokyo, Japan). For fluorescein staining, the mice corneas were divided into 3 equal upper, middle, and lower zones. Each zone had a staining score ranging between 0 and 3 points with the minimum and maximum total staining scores ranging between 0 and 9 points. The presence of scarce staining in 1 zone was scored as 1 point, whereas punctate staining covering the entire zone was scored as 3 points as previously described [12].

### Mouse meibomian gland histopathological assessment

Fresh eyelid specimens obtained from *Sod1* ^−/−^ and WT mice were fixed in 4% buffered paraformaldehyde overnight at 4°C. The tissues were embedded in paraffin, sectioned using a microtome set at 4 µm using standard techniques, and then processed for staining [12]. Morphological assessment of MG changes was performed using Hematoxylin and Eosin staining and Mallory staining for fibrosis as described previously [16], [17].

### Meibomian gland acinar unit density quantification

A photographer who was unaware of the genetic background of the mice took five representative non overlapping images from each mouse eye of the 4 groups for *Sod1^−/−^* and WT mice at 10 and 50 weeks. Each group of *Sod1^−/−^* mice at 10 and 50 weeks had 7 animals. There were 10 wild type mice at 10 and 50 weeks for meibomian gland acinar unit density quantification.

These images were taken from tissue sections cut at the same depth. Acinar units were counted manually within 445 µm×352 µm frames using an Axioplan2 Imaging microscope, Carl Zeiss, Jena, Germany. Scores from the samples were averaged as the MG acinar unit density for each group.

### Immunohistochemistry staining for CD45 panleukocyte antigen

Leucocyte common Ag (CD45) immunohistochemistry staining was performed to investigate the inflammatory changes in the MGs over time in the *Sod1^−/−^* and the WT mice. Peroxidase system Vectastain ABC kit (rat IgG; Vector Laboratories, Burlingame, CA), and anti-mouse CD45 antibody solution diluted with rabbit blocking serum at a concentration of 10 µg/mL (BioLegend, San Diego, CA) were used. Tissue sections were incubated with normal rabbit serum (Vector Laboratories, Burlingame, CA) for 2 hours at room temperature to block nonspecific background staining. The tissues were then treated with 10 µg/mL of anti-mouse CD45 for 2 hours at room temperature. For the negative controls, the primary antibody was replaced with rat IgG2B isotype control at the same concentration as the primary antibody (R&D Systems, Minneapolis, MN). Sections were then blocked using 3.0% H2O2 in methanol for 3 minutes. The tissue samples were treated with biotin-labeled rabbit anti-rat IgG serum (Vector Laboratories) for 30 minutes, followed by avidin-biotin-alkaline phosphatase complex treatment (Vector Laboratories, Burlingame, CA) for 30 minutes. The sections were then washed in 0.1M phosphate-buffered saline (PBS), developed in prepared DAB chromogen solution (Vector Laboratories, Burlingame, CA), lightly counterstained with hematoxylin for 4 minutes at room temperature, washed with tap water, dehydrated, and mounted. Sections were then evaluated and imaged using an Axioplan2 Imaging microscope, Carl Zeiss, Jena, Germany.

### Tear and serum cytometric bead array for assessment of inflammatory cytokines

Tear and serum samples were collected from 10 and 50 week mice just before sacrifice and stored at −80°C as previously reported [12]. The Becton Dickinson Cytometric Bead Array system using Bead-Based Immunoassays was applied. Mouse Th1/Th2 cytokine Kit-II (BD Bioscience, Franklin Lakes, NJ) was used to measure the levels of six inflammatory molecules, namely, interleukin (IL)-2, IL-4, IL-6, IL-10, tumor necrosis factor (TNF)-α and IFN-γ. The inflammation kit allowed detection of inflammatory cytokines in the small volumes of tear and serum samples [12]. Flow cytometric analysis was performed using a FACS Calibur flow cytometer (Becton Dickinson Immunocytometry Systems, San Jose, CA). Data were acquired and analyzed using the Becton Dickinson Cytometric Bead Array software version 1.4 (BD Bioscience) [18].

### Assessment of oxidative stress markers by immunohistochemistry

To assess the oxidative stress induced lipid peroxidation and DNA damage, anti 4-hydroxy-2-nonenal (4-HNE) and anti–8-hydroxy-2-deoxyguanosine (8-OHdG) immunohistochemistry stainings were performed (Japan Institute for the Control of Aging [JaICA], Shizuoka, Japan). The specificity and characterization of the anti 8-OHdG monoclonal antibody (N45.1) used in our study were investigated by Toyokuni S. et al. [19] Previous reports showed that 8-OHdG immunoreactivity reveals itself as a granular pattern in the cytosol and nucleus [20], [21]. The specificity of 4-HNE has also been shown to originate almost exclusively from phospholipid-bound arachidonic acid, and may be the most reliable marker of lipid peroxidation [22]. Also, 4-HNE does not recognize proteins treated with other aldehydes, such as 2-nonenal, 2-hexenal, 1-hexanal, 4-hydroxy-2-hexenal, formaldehyde, or glutaraldehyde [23]. The avidin-biotin peroxidase complex (ABC) method was used in immunostainings as previously reported [12]. Briefly, the eyelid tissues were treated with normal horse serum (Vector Laboratories, Burlingame, CA) for 2 hours at room temperature to block nonspecific background staining. Then, samples allocated to the assessment of lipid peroxidation were stained with anti-4-HNE monoclonal antibodies (at a concentration of 25 µg/mL diluted with horse blocking serum; Japan Institute for the Control of Aging, Shizuoka, Japan) for 2 hours at room temperature. Other samples were treated with mouse anti-8-OHdG monoclonal antibodies (at a concentration of 10 µg/mL, diluted with horse-blocking serum; Japan Institute for the Control of Aging, Shizuoka, Japan) for 2 hours at room temperature. For the negative controls, the primary antibody was replaced with mouse IgG1 isotype control (MOPC-21; Sigma, St. Louis, MO). Tissue samples were washed with PBS and then 3.0% H2O2 in methanol was applied for 3 minutes to block endogenous peroxidase activity. The sections were incubated for 30 minutes with biotin-labeled horse anti-mouse IgG serum (Vector Laboratories, Burlingame, CA), followed by treatment with avidin-biotin-alkaline phosphatase complex (Vector Laboratories, Burlingame, CA) for 30 minutes. All sections were washed in PBS buffer, developed in 3,3′-diaminobenzidine (DAB) chromogen solution (Vector Laboratories, Burlingame, CA). Tissues stained with anti-4-HNE antibody were lightly counterstained with hematoxylin for 4 minutes at room temperature and mounted. Using image processing software (Adobe Photoshop, San Jose, CA) a subset of colors that indicated the stained areas (brown color) was selected from the raw pictures and saved as jpeg images. Another image analysis program (Image J, NIH, Bethesda, MD) was used to measure the intensity of staining for each image, and the area of staining was calculated and expressed in pixels [12].

### Assessment of lipid staining changes in the meibomian gland

Fresh eyelids were collected from *Sod1* ^−/−^ and WT mice after sacrifice, immediately fixed with 4% paraformaldehyde and inserted in OCT, frozen (at −20°C) for staining with Oil Red O (ORO). Cryosections (5 µm thick) were then cut and mounted on glass slides for Oil Red O staining (ORO, Merck KGaA, Darmstadt, Germany). In brief, frozen sections were placed in 60% 2- propanol for 5 minutes and then stained with ORO solution for 20 min. Sections were then rinsed with PBS and counterstained with hematoxylin.

### TUNEL immunofluorescence staining for assessment of apoptosis meibomian gland acinar epithelial cells

Apoptosis immunofluorescence staining was performed using the In situ Cell Death Detection Kit, TMR Red (Roche Applied Science, Mannheim, Germany). Initially, 10 µg/ml of proteinase K (Roche Applied Science, Mannheim, Germany) in 10 mmol/L Tris/HCl (pH 7.4) was applied to the MG specimens and left for 15 minutes at room temperature. After washing the samples with 0.1M PBS twice, TUNEL reaction mixture (Roche Applied Science, Mannheim, Germany) was added on to the samples and the label solution on the negative control samples, then incubated at 37°C for 60 minutes in a dark room. The specimens were rinsed 3 times in 1M PBS for 5 minutes each, and then 100 µl of 0.5 µg/ml DAPI diluted in Tris-buffered saline and Tween-20 were added to the samples for 5 minutes at room temperature. Finally, the specimens were washed with 1M PBS and mounted with aqueous mounting medium Permafluor (Beckman Coulter, Marseille, France). Sections were examined and photographed with an epifluorescence microscope (Axioplan2 Imaging, Carl Zeiss, Jena, Germany).

### Transmission electron microscopy

Meibomian gland of 10 and 50 week old *Sod1* ^−/−^ and WT mice underwent transmission electron microscopy scanning. Meibomian gland specimens were immediately fixed after sacrifice by immersing them in 2.5% glutaraldehyde in 0.1M PBS (pH 7.4) for 4 hours at 4°C. The samples were then washed three times with 0.1M PBS solution, postfixed in 2% osmium tetroxide, dehydrated using standard series of ethanol and propylene oxide concentrations, then embedded in epoxy resin. One-micrometer sections were stained with methylene blue and MG tissues were then thin sectioned on an ultratome (LKB; Gaithersburg, MD, USA) with a diamond knife. Sections were collected on 150-mesh grids, stained with uranyl acetate and lead citrate, examined and photographed using an electron microscope (model 1200 EXII; JEOL, Tokyo, Japan).

### Statistical analysis

Data were processed using Graph Pad software (InStat, San Diego, U.S.A).

Statistical analysis was performed using the Mann-Whitney test. Differences between the data were considered significant when the *p* values were less than 0.05. The data are represented as mean values plus or minus standard deviations. Five randomly selected non-overlapping images from each eye of the *Sod1* ^−/−^ and WT mice were used for quantifying MG acinar unit density, oxidative stress markers, TUNEL and CD45 stainings. The statistician was masked from any information about the mice.

## Results

### Aqueous tear production alterations in the *Sod1* ^−/−^ and wild type mice

**Figure 1:**
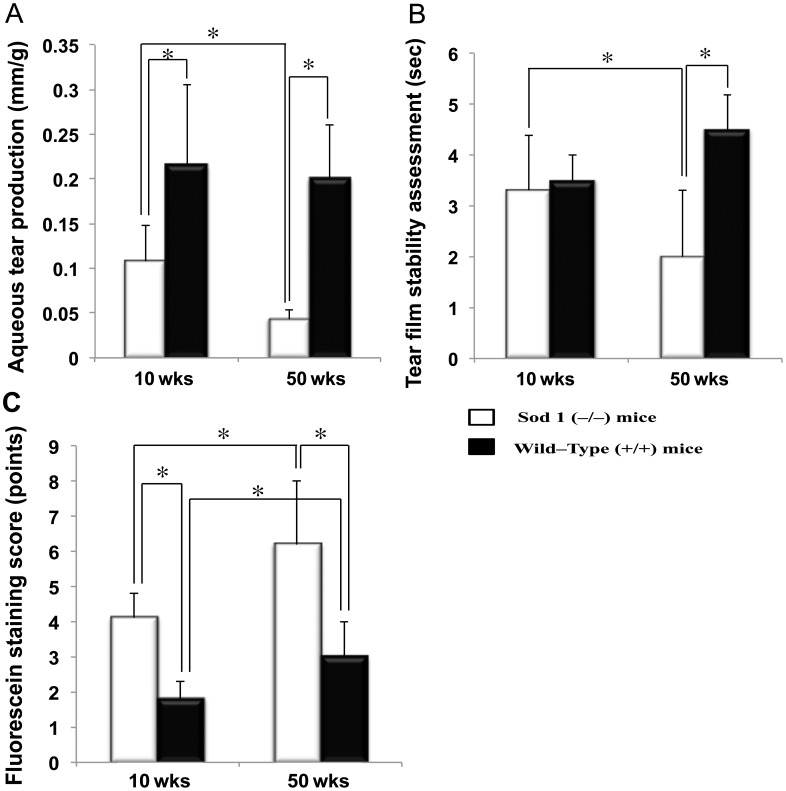
Aqueous tear production, tear stability and ocular surface epithelial cell damage assessment. A, The weight adjusted tear quantity decreased significantly from 10 to 50 weeks in the Cu, Zn-Superoxide Dismutase-1 knockout (*Sod1* ^−/−^) mice (*p* = 0.0012). The tear quantity was also significantly lower in the *Sod1* ^−/−^ mice compared to the age matched wild type (WT) mice at 10 and 50 weeks (*p* = 0.0079 and *p*<0.0001, respectively). B, The mean tear break-up time values decreased significantly from 10 to 50 weeks in the *Sod1* ^−/−^ mice (*p* = 0.0330). The tear stability was significantly worse in the *Sod1* ^−/−^ mice compared to WT mice at 50 weeks (*p* = 0.0004). C, Fluorescein staining scores were significantly higher in the *Sod1* ^−/−^ mice compared to the WT mice both at 10 (*p* = 0.0005) and 50 (*p* = 0.0006) weeks. Fluorescein staining also increased significantly from 10 to 50 weeks in both the *Sod1* ^−/−^ and WT mice (*p* = 0.0113 and *p* = 0.0032, respectively). Data represent the mean ± standard deviation of 9 mice from the *Sod1* ^−/−^ groups and 10 mice from the WT groups, at 10 and 50 weeks.

The mean weight adjusted aqueous tear production was significantly lower in the *Sod1* ^−/−^ mice compared to the WT mice at 10 and 50 weeks (*p* = 0.0079, *p*<0.0001, respectively) (Figure 1A). The tear quantity showed a decreasing tendency in the WT mice and a significant decrease was observed in the *Sod1* ^−/−^ mice from 10 to 50 weeks (*p* = 0.0012).

### Corneal vital staining and tear film stability changes

The mean tear BUT and corneal vital staining scores worsened significantly (*p* = 0.0330, *p* = 0.0113) from 10 to 50 weeks in the *Sod1* ^−/−^ mice (Figures 1B, C). Significantly lower tear BUT was observed in the *Sod1* ^−/−^ mice compared to the WT mice at 50 weeks (*p* = 0.0004) (Figure 1B). The mean corneal vital staining score was significantly worse in the *Sod1* ^−/−^ mice at 10 (*p* = 0.0005) and 50 (*p* = 0.0006) weeks compared to the age matched WT mice (Figure 1C).

### Meibomian gland alterations from 10 to 50 weeks

**Figure 2:**
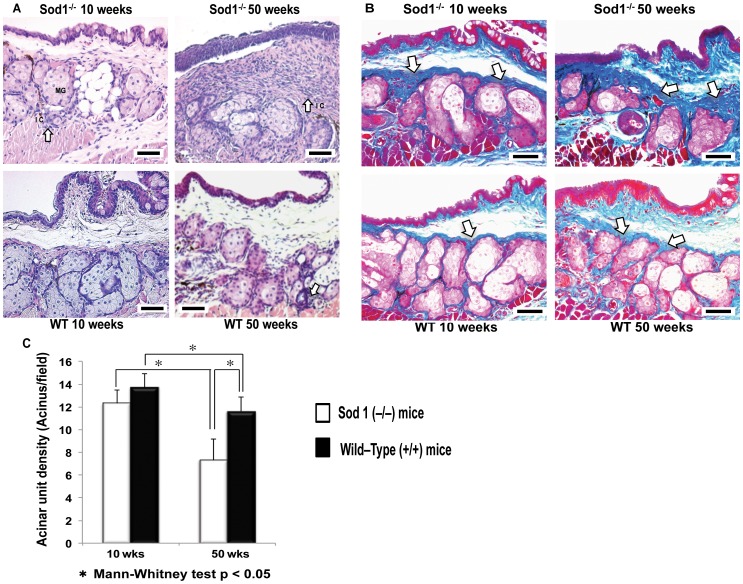
Meibomian gland histopathological alterations and tissue fibrosis in the *Sod1* ^−/−^ and wild type mice. A, Representative images for meibomian glands from the 10 and 50 week Cu, Zn-Superoxide Dismutase-1 knockout (*Sod1* ^−/−^) and wild type (WT) mice show normal meibomian gland acinar units morphology. Note the extensive periglandular inflammatory infiltration, and meibomian gland changes in the *Sod1* ^−/−^ mice at 50 weeks. Bar = 50 micrometer. B, Mallory stainings show increased fibrosis (dark blue stained areas) from 10 to 50 weeks in the *Sod1* ^−/−^ mice. Similar changes were observed in the WT mice but not to the extent observed in the *Sod1* ^−/−^ mice. Bar = 50 micrometer. C, Meibomian gland acinar unit density significantly decreased from 10 to 50 weeks in both *Sod1* ^−/−^ mice and the WT mice (*p* = 0.0007 and *p* = 0.0175, respectively). Meibomian gland acinar unit density was also significantly lower in the *Sod1* ^−/−^ mice compared to the WT mice at 50 weeks (*p* = 0.0003). Five tissue sections of each mouse eye were analyzed to produce the meibomian gland acinar unit density values. Data represent the mean ± standard deviation for at least 7 mice from the *Sod1* ^−/−^ groups and 10 mice from the WT groups, at 10 and 50 weeks. Five tissues sections from each eye of 7 animals (5 images per animal's eye) for each group were analyzed to produce the representative Mallory staining images.

Hematoxylin and Eosin staining showed an increase in periglandular inflammatory infiltrates, a decrease in MG glandular acinar density and an increase in periglandular fibrosis in the *Sod1* ^−/−^ and WT mice from 10 to 50 weeks (Figure 2A). At 10 weeks, *Sod1* ^−/−^ and WT mice showed normal morphology and density for the MG acinar units. Fifty week old *Sod1* ^−/−^ mice developed a severe inflammatory response compared to age matched WT mice, with the inflammatory cells invading the periglandular spaces (Figure 2A). Quantification of the MG acinar units in the upper lid samples showed no statistically significant differences between the mean acinar units densities of *Sod1* ^−/−^ (12.3±1.2 acinus/field) and WT mice (13.7±1.2 acinus/field) at 10 weeks (*p* = 0.0628). The mean acinar unit densities in the MG specimens for the *Sod1* ^−/−^ mice at 50 weeks (7.3±1.9 acinus/field) were significantly lower than the densities in the age matched WT mice (11.6±1.3 acinus/field) (*p* = 0.0003) (Figure 2C). A similar trend was observed in the lower lid samples of *Sod1* ^−/−^ and age matched WT mice (data not shown).

Mallory staining was more dense in the MG specimens of all *Sod1* ^−/−^ mice at 50 weeks indicating a higher extent of fibrosis. We observed dense periglandular staining in the 50 week *Sod1* ^−/−^ mice with some slight interacinar positive staining in the age matched WT mice (Figure 2B).

### Changes in tear, serum and eye lid tissue inflammatory markers

**Figure 3:**
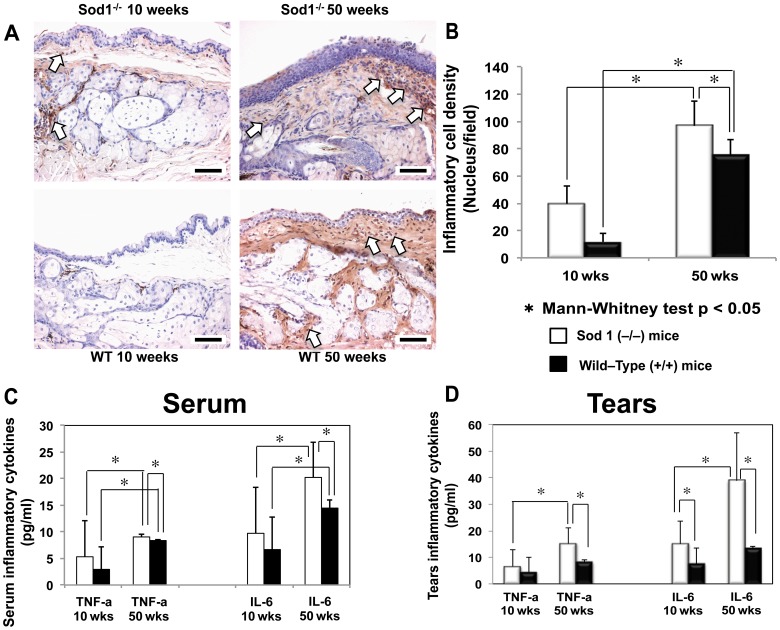
Inflammatory changes in the meibomian glands, serum and tears of the *Sod1* ^−/−^ and wild type mice. A, Specimens stained with CD45 from 10 and 50 weeks Cu, Zn-Superoxide Dismutase-1 knockout (*Sod1* ^−/−^) and wild type (WT) mice. Note the timewise increase in inflammatory cell staining from 10 to 50 weeks in the *Sod1* ^−/−^ mice. Wild type mice specimens showed scant inflammatory cell staining, which increased significantly in the 50 week WT mice but not to the extent observed in the *Sod1* ^−/−^ mice at 50 weeks. Bar = 50 micrometer. B, A significant timewise increase in the mean inflammatory cell densities from 10 to 50 weeks was observed in both the *Sod1* ^−/−^ and WT mice (*p* = 0.0143 and *p* = 0.0286, respectively). Note the significantly higher inflammatory cell density in the *Sod1* ^−/−^ mice at 50 weeks compared to the age matched WT mice (*p* = 0.0317). Five tissue sections of each eye of 6 animals (5 per mouse eye) were analyzed to produce the figure. Data represent the mean ± standard deviation for 6 mice from the *Sod1* ^−/−^ groups and 6 mice from the WT groups at 10 and 50 weeks. C, The mean serum IL-6 concentration increased significantly from 10 to 50 weeks in both the *Sod1* ^−/−^ (*p* = 0.0195) and WT mice (*p* = 0.0001). Serum IL-6 concentration was significantly higher in the 50 week *Sod1* ^−/−^ compared to 50 week WT mice (*p* = 0.0100). Serum TNF-α levels were also significantly higher (*p* = 0.0011) in the 50 week *Sod1* ^−/−^ mice compared to the age matched WT mice. The mean serum TNF-α concentration significantly increased from 10 to 50 weeks in the *Sod1* ^−/−^ mice (*p* = 0.0457). Data represent the mean ± standard deviation for 14 and 8 *Sod1* ^−/−^ mice at 10 and 50 weeks as well as 11 and 9 wild type mice at 10 and 50 weeks, respectively. D, There was also a significant (*p* = 0.0148) timewise increase in the mean tear IL-6 concentration in the *Sod1* ^−/−^ mice from 10 to 50 weeks. Note the significantly higher IL-6 concentration in the *Sod1* ^−/−^ mice at 10 (*p* = 0.0414) and 50 weeks (*p* = 0.0022) compared to the age matched WT mice. Tear TNF-α concentrations increased significantly from 10 to 50 weeks in the *Sod1* ^−/−^ (*p* = 0.0115). Also note the significantly higher TNF-α concentration in the *Sod1* ^−/−^ mice compared to the WT mice at 50 weeks (*p* = 0.0087). Data represent the mean ± standard deviation for 7 mice from the *Sod1* ^−/−^ groups and 9 mice from the WT groups at 10 and 50 weeks.

To assess the eye lid inflammation status, we conducted CD45 panleukocyte marker staining [24]. CD45 antibodies showed scant staining of the inflammatory cells with anti CD45 antibodies in the 10 week *Sod1* ^−/−^ and WT mice (Figure 3A). However, we observed intense periglandular staining in the *Sod1* ^−/−^ mice at 50 weeks, with a higher number of stained cells than those observed in the 50 week WT mice (Figure 3A). We then quantified the total inflammatory cell counts in each specimen using the ImageJ (A Java software program developed by the National Institutes of Health, Bethesda, MD) and Adobe Photoshop. A significant increase in the mean inflammatory cell densities from 10 weeks to 50 weeks both in the *Sod1* ^−/−^ and WT mice was observed (*p* = 0.0143 and *p* = 0.0286, respectively) (Figure 3B). The mean inflammatory cell density was significantly higher in the *Sod1* ^−/−^ mice compared to the WT mice at 50 weeks (*p* = 0.0317) (Figure 3B).

Evaluation of inflammatory cytokine concentrations in the serum and tears, revealed a significant increase in the mean serum IL-6 concentration in the *Sod1* ^−/−^ mice from 10 to 50 weeks (*p* = 0.0195) (Figure 3C). Serum TNF-α levels were also significantly higher (*p* = 0.0011) in the *Sod1* ^−/−^ mice (9.13±0.42 pg/ml) compared to the WT mice at 50 weeks (8.30±0.28 pg/ml) (Figure 3C). There was a significant (*p* = 0.0457) increase in the mean TNF-α serum concentration from 10 weeks (5.26±6.84 pg/ml) to 50 weeks (9.13±0.42 pg/ml) in the *Sod1* ^−/−^ mice (Figure 3C).

The mean tear IL-6 concentration also showed a significant increase in the *Sod1* ^−/−^ mice from 10 to 50 weeks (*p* = 0.0148) (Figure 3D). The IL-6 concentration was significantly higher (*p* = 0.0022) in the *Sod1* ^−/−^ at 50 weeks (38.80±18.00 pg/ml) compared to the WT mice at 50 weeks (13.29±0.82 pg/ml) (Figure 3D). The mean tear TNF-α concentrations increased significantly (*p* = 0.0115) from 10 to 50 weeks in the *Sod1* ^−/−^ (6.46±6.57 pg/ml and 15.20±6.08 pg/ml, respectively). The WT mice levels showed a tendency to increase but without significance (*p* = 0.1405) from 10 to 50 weeks (4.41±5.78 pg/ml and 8.04±1.09 pg/ml, respectively). The mean tear TNF-α concentration was significantly higher in the *Sod1* ^−/−^ mice compared to the WT mice at 50 weeks (*p* = 0.0087) (Figure 3D). The mean tear and serum IL-2, IL-4, IL-10 and IFN-γ concentrations did not reveal statistically significant differences from 10 to 50 weeks in both the *Sod1* ^−/−^ and WT mice. There were also no significant differences in the cytokine concentrations between the *Sod1* ^−/−^ and WT mice at 50 weeks (data not shown).

### Changes in oxidative stress markers in the meibomian glands

**Figure 4:**
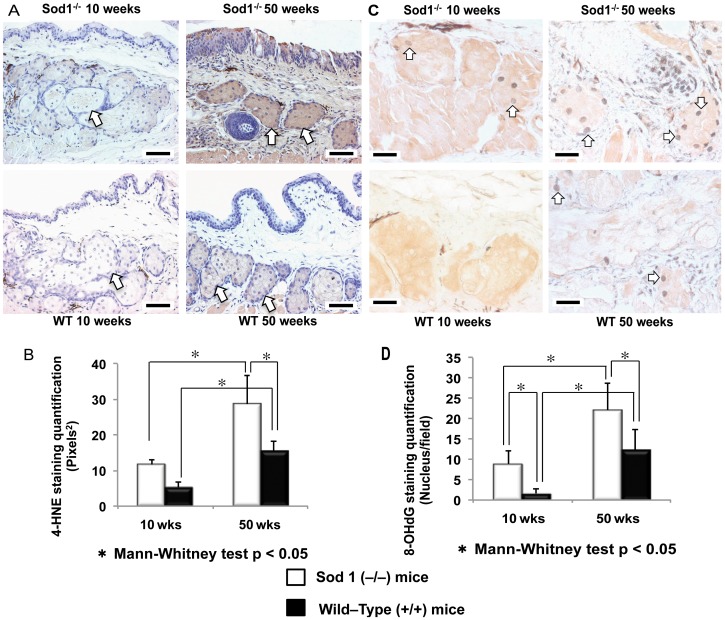
Oxidative lipid and DNA changes in the meibomian glands. A, Late phase lipid peroxidation marker 4-HNE stainings show dense staining in the 50 week old Cu, Zn-Superoxide Dismutase-1 knockout (*Sod1* ^−/−^) mice. Wild type (WT) mice specimens also had some staining but not to the extent observed in the same age *Sod1* ^−/−^ mice. Bar = 50 micrometer. B, The extent of cellular staining with 4-HNE was significantly higher in the *Sod1* ^−/−^ than the WT mice at 50 weeks (*p* = 0.0022). Note the significant timewise elevation in the anti-4-HNE staining from 10 to 50 weeks in the *Sod1* ^−/−^ mice. C, Staining with 8-OHdG antibodies in *Sod1* ^−/−^ and WT mice samples at 10 and 50 weeks. Meibomian gland acinar cell nuclei showed scant staining in 10 week *Sod1* ^−/−^ and WT mice. Bar = 100 micrometer. There was a marked increase in nuclear staining from 10 to 50 weeks, especially in *Sod1* ^−/−^ mice. Relatively more acinar nuclei were stained with anti-8-OHdG antibodies in the *Sod1* ^−/−^ mice at 50 weeks compared to meibomian gland specimens from the same age WT mice. D, Quantitative assessment for the cellular staining of anti-8-OHdG antibodies showed a statistically significant timewise increase from 10 to 50 weeks in *Sod1* ^−/−^ and WT mice (*p* = 0.0003, *p* = 0.0011, respectively). A significant timewise elevation in staining was observed in the *Sod1* ^−/−^ and WT mice at 50 weeks (*p* = 0.0133). Five tissue sections of each animal's eye were analyzed to produce the figure. Data represent the mean ± standard deviation for 7 mice from the *Sod1* ^−/−^ group and 6 mice from the wild type group at 10 and 50 weeks.

To evaluate the influence of the oxidative stress on the lipid peroxidation markers, we initially performed MG immunohistochemistry stainings with anti 4-HNE antibodies (Figure 4A). Extensively dense staining was observed in the specimens from the 50 week old *Sod1* ^−/−^ mice compared to the those from the age matched WT mice and 10 week old *Sod1* ^−/−^ and WT mice (Figure 4A). The mean area (pixels^2^) of positively stained cells was 11.6±1.4 for *Sod1* ^−/−^ mice at 10 weeks, 5.3±1.4 for WT mice at 10 weeks, 15.4±2.8 for WT mice at 50 weeks and 28.7±8.1 for *Sod1* ^−/−^ mice at 50 weeks as shown in figure 4B. Meibomian gland 4-HNE staining significantly increased in the *Sod1* ^−/−^ (*p* = 0.0119) and WT (*p* = 0.0079) mice from 10 weeks to 50 weeks. The extent of 4-HNE antibody staining in the *Sod1^−/−^* mice at 50 weeks was significantly higher (*p* = 0.0022) than the staining in the 50 week WT mice (Figure 4B).

We also observed a marked increase in staining with anti-8-OHdG antibodies in MG nuclei from 10 to 50 weeks, in all *Sod1* ^−/−^ mice (Figure 4C). Significantly higher acinar epithelial cell nuclear staining with anti-8-OHdG antibodies in the *Sod1* ^−/−^ mice was found at 50 weeks compared with MG specimens taken from the WT mice at 50 weeks (*p* = 0.0133), as shown in Figure 4D.

### Evaluation of meibomian gland lipid staining alterations in the *Sod1* ^−/−^ and wild type mice

**Figure 5:**
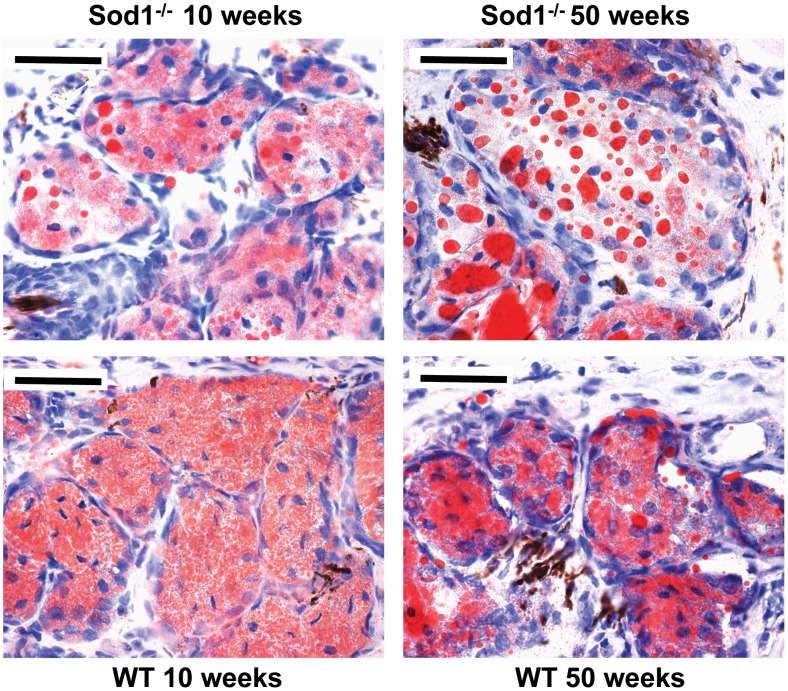
Timewise alterations in meibomian gland lipid staining. Oil Red O staining showed an accumulation of large lipid droplets in the Cu, Zn-Superoxide Dismutase-1 knockout (*Sod1* ^−/−^) mice from 10 to 50 weeks. Note the diffusely uniform staining pattern in the wild type (WT) mice at 10 weeks and some accumulation of large lipid droplets in the WT mice at 50 weeks, but not to the extent observed in the same age *Sod1* ^−/−^ mice. Bar = 100 micrometer. Five tissues sections from each eye of the 7 animals in each mice group were analyzed to produce the representative images.

Oil Red O staining showed an accumulation of large lipid droplets in the acinar units of the 50 week *Sod1* ^−/−^ mice. The lipid droplets appeared to become larger in size and increased in number from 10 to 50 weeks (Figure 5). There was a diffusely uniform staining pattern in the 10 week old WT mice. A few large lipid droplets were observed in the WT mice at 50 weeks but not to the extent observed in the *Sod1* ^−/−^ mice.

### Changes in apoptosis within the meibomian glands

**Figure 6:**
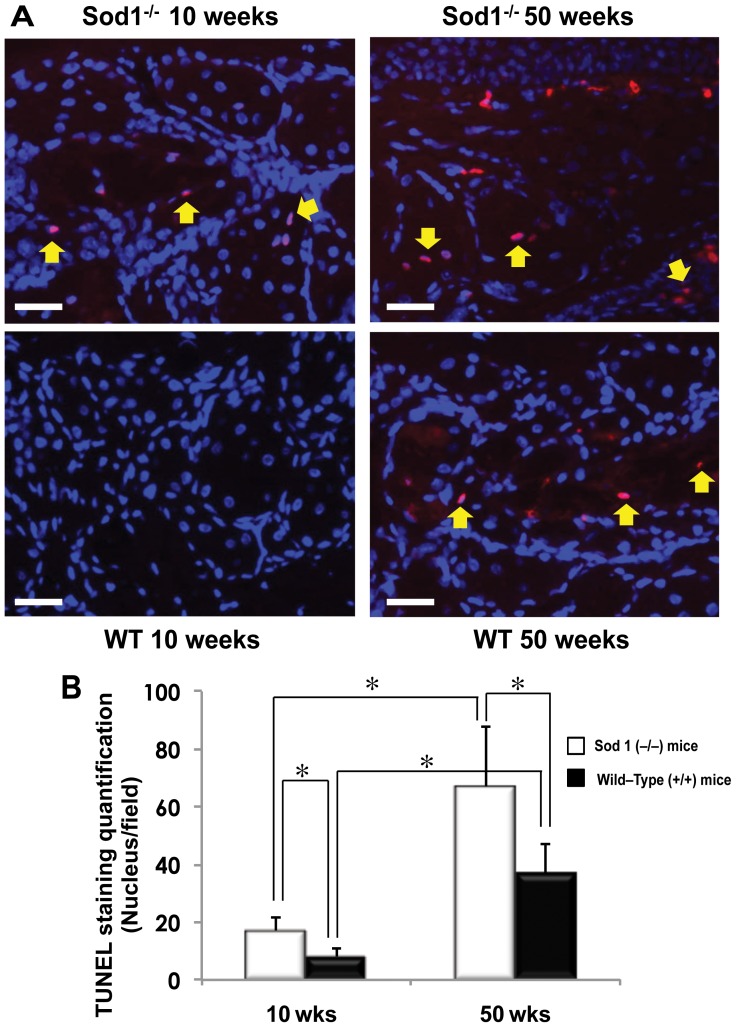
Immunohistochemical and quantitative evaluation of the timewise apoptotic changes in meibomian glands. A, Meibomian gland samples from Cu, Zn-Superoxide Dismutase-1 knockout (*Sod1* ^−/−^) and wild type (WT) mice at 10 and 50 weeks stained with TUNEL (red) and DAPI (blue). There was marked positive staining with TUNEL for apoptotic cells in the 50 week *Sod1* ^−/−^ mice in comparison to specimens from *Sod1* ^−/−^ mice at 10 weeks and WT mice at 50 weeks. Yellow arrows indicate cells positively stained with TUNEL. A timewise increase in positively stained cells was also observed in WT mice meibomian gland specimens from 10 weeks to 50 weeks. Bar = 100 micrometer. B, There was a statistically significant difference between the number of positively stained cell densities of the *Sod1* ^−/−^ and WT mice at 10 weeks (*p* = 0.0006). A significant timewise increase in the number of positively stained cell densities from 10 to 50 weeks was observed in both *Sod1* ^−/−^ and WT mice (*p*<0.0001). The density of positively stained cells in the meibomian gland specimens of the *Sod1* ^−/−^ mice at 50 weeks was significantly higher than for those observed in the 50 week WT mice (*p*<0.0001). The images represent at least five independent samples of mouse eye per group. Data represent the mean ± standard deviation for 7 mice from the *Sod1* ^−/−^ groups and 6 mice from the WT groups at 10 and 50 weeks.

Immunofluorescence staining with TUNEL assay (terminal deoxyribonucleotidyl transferase [TdT]-mediated deoxyuridine triphosphate [dUTP]-digoxigenin nick end labeling) was used to detect the DNA breakpoints and assess the presence of apoptotic cells (Figure 6A). MG samples in the 50 week old *Sod1* ^−/−^ mice showed marked positive staining with TUNEL for apoptotic cells (67.1±20.7 nuclei/field) in comparison to specimens from the *Sod1* ^−/−^ mice at 10 weeks (16.9±4.6 nuclei/field) (*p*<0.0001) and the WT mice at 50 weeks (37.1±9.9 nuclei/field) (*p*<0.0001) (Figure 6B). We also observed significantly increased positive staining in the MG specimens (*p*<0.0001) of the WT mice from 10 weeks (7.8±3.1 nuclei/field) to 50 weeks (37.1±9.9 nuclei/field).

### Ultrastructural mitochondrial alterations

**Figure 7:**
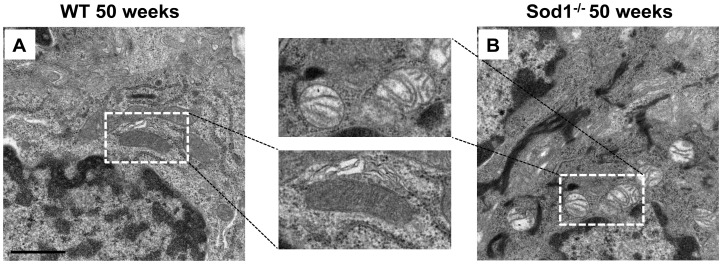
Transmission electron microscopic examination of the mitochondrial alterations. Marked ultrastructural changes in the mitochondria including swelling, disorientation, shortening, and disorganization of cristae were noted at 50 weeks in the Cu, Zn-Superoxide Dismutase-1 knockout (*Sod1* ^−/−^) mice. We observed abnormalities in 40 percent of the 50 week wild type (WT) mice and 80 percent in the age matched *Sod1* ^−/−^ mice. No such phenotypic alterations were observed in the mitochondria of the 10 to 50 week WT mice. Bar = 1 micrometer. Five tissues sections from each eye of the 6 animals in each mice group were analyzed to produce the representative images.

Using transmission electron microscopy we observed ultrastructural changes in the mitochondria of the *Sod1* ^−/−^ mice at 50 weeks, including mitochondrial swelling, disorientation, shortening, and disorganization of cristae compared to age matched WT mice (Figure 7A, B). No specific phenotypic alterations in the mitochondria of WT or the *Sod1* ^−/−^ mice at 10 weeks were observed (Data not shown). We detected mitochondrial abnormalities in 40 percent of the 50 week WT mice samples and 80 percent in the age matched *Sod1* ^−/−^ mice samples.

## Discussion

This study showed the presence of histopathological, functional and inflammatory age dependent changes in the MG of WT mice that were accelerated in *Sod1* ^−/−^ mice. We observed a significant decrease in aqueous tear production, increased ocular surface epithelial cell damage and elevated inflammatory cytokines levels in tear and serum of *Sod1* ^−/−^ mice compared to WT mice at 50 weeks. In comparison to the WT mice, histopathological investigations of the *Sod1* ^−/−^ mice from 10 to 50 weeks showed greater deleterious structural changes, increases in lipid and DNA oxidative damage, elevated apoptosis, as well as changes in the mitochondrial ultrastructure. These results suggest that the *Sod1* ^−/−^ mice experience many of the biochemical and structural changes found in MGD, suggesting a role for oxidative stress in this pathology.

Exposure to exogenous and endogenous ROS leads to cellular death and degeneration [25]. Elevated oxidative stress has been reported to play a role in many systemic diseases such as Alzheimer's disease [26], [27], Parkinson's disease, cancer [28], [29], [30] and ischemic disorders, due to oxygen reperfusion injury followed by hypoxia [31], [32]. In the field of ocular diseases, oxidative stress has been reported to be involved in the development of age-related macular degeneration [11], cataract [33], keratitis [34], uveitis [35], and dry eye disease [36]. However, the role of oxidative stress in the pathogenesis of MGD has not been investigated in the *Sod1* ^−/−^ model as yet.

Cu,Zn-SOD1enzyme has been identified as a soluble enzyme widely distributed in the cytoplasm of all mammalian cells [37]. It has the highest amount and activity among the superoxide dismutase enzymes family [37]. Thus, enzyme dismutes O_2_-provide essential protection for biological tissues against uncontrolled reactions with oxygen-based radicals.

It has been reported that Cu, Zn-SOD1-knockout (*Sod1* ^−/−^) leads to aggravation of oxidative stress status resulting in various aging phenotypes including skin and muscle atrophy [38], [39], fatty liver deposits [16], and macular degeneration [11].

To clarify the pathogenesis of MGD, we investigated the histopathological alterations and oxidative stress related changes in the MG using *Sod1* ^−/−^ mice. We conducted stainings using 4-HNE, a well recognized and studied cytotoxic product of lipid peroxidation, and also 8-OHdG DNA oxidative stress marker [40]. We noted extensive oxidative stress related lipid and DNA damage in the MG epithelia, which seemed to increase with aging from 10 to 50 weeks in both the *Sod1* ^−/−^ and WT mice. The lipid and DNA damage appeared to be more extensive in the 50 week *Sod1* ^−/−^ mice compared to the same age WT mice. This is consistent with our previous report that showed elevated age related lipid and DNA oxidative stress damage in the lacrimal glands of *Sod1* ^−/−^ mice in association with elevated serum 8-OHdG concentrations [12]. These changes suggest the accelerated oxidative lipid and DNA damage in the MG tissues of *Sod1* ^−/−^ mice may be the result of the accumulation of ROS due to the lack of SOD1 enzyme protection.

Oxidative damage has also been reported to be associated with induction of inflammation [41], [42], [43]. CD45 is a panleukocyte marker that has been shown to be a good marker for staining of T lymphocytes, B lymphocytes, granulocytes, monocytes, and macrophages [24]. Our histopathological analysis with CD45 stainings showed a time related increase in meibomian periglandular inflammatory infiltrates. This was associated with a decrease in MG acinar density and an increase in periglandular fibrosis in the *Sod1* ^−/−^ and WT mice from 10 to 50 weeks. These changes were more prominent in the *Sod1* ^−/−^ mice. Our findings were consistent with previous studies that showed increased periglandular infiltrates in the MG tissues of aging mice and humans [13], [44]. Obata et al reported similar atrophy of the acini and the presence of granulated tissue in the MGs of postmortem samples taken from an elderly population [6].

The presence of inflammatory cells, in addition to elevated levels of the inflammatory markers TNF-α and IL-6 in the tear and serum samples of the *Sod1* ^−/−^ mice compared to WT mice from 10 to 50 weeks suggests the presence of a systemic inflammatory status. In our believe SOD1 enzyme knockout resulted in accumulation of ROS and induced oxidative stress. Indeed, elevated oxidative stress has been shown to promote inflammation by activating the redox-sensitive transcription factor, nuclear factor-kappa B (NF-κ B), which, in turn, triggers generation of pro-inflammatory cytokines and chemokines, and hence, inflammation [45], [46]. While we have not investigated specific markers involved in NF-κ B pathway activation in this study, we believe that future studies should undertake this. These studies should also investigate the role of MAP kinase pathways. Increased TNF-α and IL-6 concentrations have also been reported in dry eye syndromes [47], [48], [49], [50], [51], [52]. TNF-α has been reported to play an important role in inflammation and cell death, while IL-6 has been connected with inflammation and fibrosis [53], [54], [55], [56], [57]. Such inflammatory and fibrotic changes have been reported to be associated with increases in the incidence of cellular death and apoptosis [58].

A noteworthy and interesting observation was the accumulation of secreted lipids in the MGs. Oil Red O staining showed an abundance of large lipid droplets in the MG acinar units of the 50 week *Sod1* ^−/−^ mice, unlike the diffusely uniform staining patterns observed in the younger mice. These observations may be attributed to the changes in the composition and volume of the lipid content. The possible decrease in lipid secretion could be due to the decline in lipid producing acinar tissues in the older mice and the inability to secrete the lipid onto the ocular surface in the *Sod1* ^−/−^ mice. These results are similar to a previous report that showed more intense ORO staining in the MGs from older mice [13]. The same report showed that age-related changes in the localization of the peroxisome proliferator activator receptor (PPARγ) was associated with changes in the lipid production as well as the size of the MG in mice. Another study by Uchiyama et al. showed enhanced lipid accumulation in the liver of the *Sod1* ^−/−^ mice [16]. In humans, Sullivan et al, reported that aging is associated with numerous significant alterations in the lipid profiles of MG secretions [5]. We therefore believe that a detailed investigation of the changes in the composition of the meibum in SOD1 knockout mice is necessary.

Significant deterioration in the mean tear BUT values in the *Sod1* ^−/−^ mice compared to the WT mice at 50 weeks also indicates a possible change in the composition of the tear film. This could be due to changes in the composition of the lipid and weight adjusted aqueous layers of the *Sod1* ^−/−^ mice tear film due to MG lipid changes and a decrease in the aqueous tear production. The aqueous tear production decrease observed in this study was consistent with our previous report, and can be attributed to lacrimal gland atrophy and secretory dysfunction [12]. Furthermore, tear film BUT has been reported to decrease with age in humans [5], [59].

The changes in tear film quality and quantity observed in the *Sod1* ^−/−^ mice can explain in part, the marked increase in the ocular surface damage as evidenced by the higher fluorescein staining scores in the SOD1 knockout mice.

The accumulation of reactive oxygen species has been reported to be associated with the mitochondrial alterations observed in humans and animal models of age-related diseases [27], [60], [61], [62]. Electron microscopy showed mitochondrial architectural alterations in the *Sod1* ^−/−^ mice, similar to those reported in human age related diseases [27]. We believe that mitochondrial abnormalities might lead to a decrease in ATP [63] levels in the MGs and consequently effected glandular secretions, which would then induce MGD and dry eye disease. These mitochondrial alterations have been reported to be associated with the activation of the apoptotic signals initiating cell death [64], [65], [66], [67]. The TUNEL assay used to detect the DNA breakpoints and assess apoptotic cells [68] in this study showed a significant increase of positively stained cells in the old *Sod1* ^−/−^ and wild type mice. These changes were significantly more severe in the aged *Sod1* ^−/−^ mice than in the WT mice, indicating that the accumulation of ROS plays a key role in accelerating cell death. Additionally, apoptosis of the MG acini along with glandular atrophy have been previously proposed as a possible mechanism for the impairment of glandular secretory function [6].

The reason of increased programmed cell death in *Sod1* ^−/−^ mice can be attributed in part to the increase in apoptosis induced by cytokines such as IL-6, as well as mitochondrial ultrastructural changes and decreased ATP. Although it is not fully established for all cases, it has been reported that the major driving force behind the cell death process, and very possibly many other destructive processes, is the generation of free radicals [69].

Previous studies conducted on drug induced MGD in animal models, including rabbits [70] and monkeys [71], showed hyperkeratinization of the ductal epithelium [2], [6]. However, the mechanism of the development of MGD induced in these animals is not known. Hyperkeratinization of the MG ductal epithelium and dysfunction of the acinar cells could be another possible reason for MGD. Although we observed age dependent keratinization changes in the MG ductal epithelium of *Sod1* ^−/−^ mice (data not shown), it was not observed consistently in all samples. Further studies on age dependent MG ductal changes and the role of hyperkeratinization in the pathophysiology of MGD are definitely essential.

We believe that oxidative stress might be an integral and primary cause of age-dependent MGD and dry eye disease in the *Sod1* ^−/−^ mice model. Although our study suggests that *Sod1* ^−/−^ mice experience many of the biochemical and structural changes found in MGD, it is difficult to know if any or all of the effects seen are a result of systemic causes. In addition, the role that inflammation plays is likely to be just as important as the effect of oxidative stress and may compound the damage. The age of mice investigated in this study were 2.5 and 12.5 months, which may be considered relatively young. Future research on MGD in more elderly mice may provide a better understanding of the mechanisms of the disease. More research is essential if suitable treatments, which might include antioxidants, are to be developed the future.

In conclusion, it was demonstrated that a lack of the SOD1 enzyme leads to increased oxidative lipid and DNA damage, and an increased inflammatory status in the MG, tears and serum, inducing marked morphological alterations in the MGs of the current mouse model, which result in dry eyes and ocular surface disease. *Sod1* ^−/−^ mice may turn out to be a useful model that will help enrich our understanding of age related dry eye disease in humans.
